# Utilization of dental services of older persons after onset of home care ­­– an observational study from the InSEMaP research project based on German insurance claims data

**DOI:** 10.1186/s12877-025-06420-8

**Published:** 2025-10-15

**Authors:** Espen Henken, Hans-Helmut König, Alexander Konnopka, Anja Behrens-Potratz, Stefanie Schellhammer, Petra Schmage, Thomas Zimmermann, Claudia Konnopka

**Affiliations:** 1 Department of Health Economics and Health Services Research, University Medical Center Hamburg-Eppendorf, Martinistraße 52, Hamburg, 20246 Germany; 2 Department of Psychiatry and Psychotherapy, University of Leipzig Medical Center, Semmelweisstraße 10, Leipzig, 04103 Germany; 3 Faculty of Business and Social Sciences, Department of Nursing and Management, Cooperative Process Management in Social and Healthcare RTC (KoPM-Zentrum), Hamburg University of Applied Sciences, Alexanderstraße 1, Hamburg, 20099 Germany; 4 Department of Health Care Research and Innovation, Deutsche Angestellten-Krankenkasse-Gesundheit (DAK-G), Nagelsweg 27, Hamburg, 20097 Germany; 5 Department of Periodontics, Preventive and Restorative Dentistry, University Medical Center Hamburg-Eppendorf, Martinistraße 52, Hamburg, 20246 Germany; 6 Department of General Practice and Primary Care, University Medical Center Hamburg-Eppendorf, Martinistraße 52, Hamburg, 20246 Germany

**Keywords:** Claims data, Geriatric dentistry, Health service research, Home care, Long-term care

## Abstract

**Background:**

Maintaining a good oral health is important for general health, nutrition, and quality of life. Nevertheless, the utilization of dental services decreases with age. Persons in need of home care, in particular, might face difficulties organizing dental visits due to a decreasing functional status. The aim of this study was to analyze the utilization of dental services of older persons after the onset of need for home care.

**Methods:**

In a retrospective cohort study with statutory health insurance claims data from 2015–2020, we compared persons aged ≥ 60 years with incident and lasting need for home care in 2017 (study group) with a control group without need for care. Both groups had at least one routine dental service during the preceding 2-year baseline period. We applied entropy balancing to adjust for differences in patient characteristics. We analyzed the utilization of dental services over a 3-year follow-up. We analyzed the probability of utilization and the number of dental services using weighted logistic and negative binomial regressions.

**Results:**

We identified 26,818 persons in the study and 393,540 in the control group. We obtained a lower probability for any dental service use in the study group (0.9) than the control group (0.94; risk ratio: 0.96; 98.75% confidence interval: 0.96–0.97). Moreover, persons in the study group utilized on average fewer dental services than those in the control group (rate ratio: 0.92; 98.75% confidence interval: 0.91–0.93).

**Conclusion:**

The onset of need for home care was associated with a decreased utilization of dental services for older persons with prior dental service utilization, expanding existing evidence. There is a need to improve oral healthcare for persons in need of home care.

**Trial registration:**

The study was included in the German Clinical Trials Register (DRKS) on 04/01/2022: DRKS00027020. https://drks.de/search/de/trial/DRKS00027020

**Supplementary Information:**

The online version contains supplementary material available at 10.1186/s12877-025-06420-8.

## Background

The proportion of older persons is increasing in many countries [1]. In Germany, for example, the number of inhabitants older than 67 years will increase from 16.4 million in 2021 to at least 20.4 million at the end of the 2030s [2]. While oral health of old persons often is poor [3, 4], studies from many countries showed that utilization of dental services decreases with increasing age [5–9]. Nevertheless, regular dental visits are an important part of oral health prevention [10–13]. Research also suggests that for very old persons the motivation for seeing a dentist becomes symptom rather than check-up-driven [11]. However, oral health is important for the quality of life [14], nutrition [15], and general health [16].

An increase in the proportion of older persons will also increase the number of persons in need of long-term care, particularly the number of persons in need of home care as most care-dependent persons are cared for at home (> 80% in Germany in 2023 [17]). Persons in need of home care require help in activities of daily living due to a functional decline that leads to a loss of autonomy, but still dwell in the community. Care dependency is typically associated with a high rate of multimorbidity and thus medical needs. To illustrate, a claims data analysis showed that persons in need of home care had a median of 6 chronic diseases [18].

While in Germany, nursing homes can organize dental treatments via individual cooperation-contracts with dentists [19, 20], persons in home care face particular barriers when trying to go to the dentist as their mobility may be limited. Outreach care, however, is difficult, time-consuming [21], and, as argued in two recent studies, reimbursement for outreach care in Germany might be too low for dentists to be attractive [5, 22]. In line, a systematic review on care dependent persons in general described a lack of suitable facilities for treatment or transportation of the patient as one of the most common barriers to provide dental treatment for care dependent older persons [21]. Thus, depending on the level of functional abilities, it might be necessary for the caregiver to organize dental visits and relating transportation. Moreover, a symptom-oriented usage of dental services by care-dependent persons increases the responsibility for caregivers to pay attention to prevention [23]. Hence, constraints in knowledge or resources of the caregivers might hinder dental service utilization of persons in need of home care.

Several investigations showed that being care-dependent was associated with a low utilization of dental services [22, 24–28] and oral health [12, 25, 26, 29, 30]. An analysis of German health insurance claims data found that 51.9% of home care recipients but 73.2% of independent older adults utilized any dental service within one year [22] even though the recommendation is one [31] or even two [32] check-up visits per year. Notably, recent German studies on persons with long-term care need have indicated that dental service utilization [22] and oral health [33] were particularly low for home care recipients. As this might be associated with the cooperation-contracts of dentists and nursing homes [34], the situation might be different in other countries (e.g., for reviews on oral health of persons in home care or nursing homes see: [30, 35]). By now, little is known about how the *onset* of a home care need affects the dental service utilization. First descriptive results from Czwikla et al. [22] showed that fewer persons saw a dentist in the year after (55.6%) compared to the year before transitioning to home care (60%). However, to investigate the impact of the onset of a need for home care on the utilization of dental services, it is important to investigate a longer period considering that dental care might not have a high priority in the year of transitioning to long-term care. Moreover, investigating the discontinuation of a regular utilization is important to evaluate the effect of the onset of need for care on oral check-up behavior.

The aim of this study was to analyze the utilization and discontinuation of dental services after the onset of need for long-term home care in comparison to persons without need for care. We hypothesized that persons with incident need for home care differed in the utilization and discontinuation of dental services from those without care need. Because many studies found regional variations in the utilization of dental services [5, 22, 36], we established as a secondary objective before data analysis (not part of the published study protocol [37]), to investigate whether the impact of an onset of a need for home care on the utilization of dental services differed with regard to population density.

## Methods

The study was part of the InSEMaP (Interaction of Systemic Morbidity and Oral Health in Ambulatory Patients in Need of Home Care) project. The reporting of this article was guided by the RECORD statement [38]. We conducted a retrospective cohort study based on health insurance and long-term care claims data from 2015 to 2020 provided by the DAK-G, one of the largest statutory health insurance companies in Germany covering 5,5 million persons [39] and thus about 6.6% of the German population [40]. We compared the utilization of dental services in a study group of persons with a lasting need for home care with a control group that never needed long-term care.

German statutory health insurances cover medically necessary dental treatments which include prevention, early detection, and treatment; tooth replacements are paid partly [41]. In Germany, need for long-term care is assessed in a standardized examination. Care recipients are categorized into five care levels depending on their functional status and ability of performing activities of daily living. According to § 15 of the German social law book (SGB XI) the care levels describe: minor (level 1), considerable (level 2), severe (level 3), and severest (level 4) impairment of independence or abilities. Level 5 describes severest impairment of independence or abilities with special care requirements. Care services are provided within a nursing home (inpatient care) or at the recipients’ home (home care). In the current study, need for home care was defined as a continuously recorded care level starting in 2017 without use of inpatient care services (except short-term care). The date of the first recorded care level was used as *index date* in the study group. Based on the index date, we used the two preceding years as baseline and the three subsequent years as follow-up periods, creating an individual observation period per person.

We included all continuously insured persons aged ≥ 60 years on December 31, 2017. We only selected persons with at least one recorded *routine dental service* in the baseline period and without care level until 2017. In the current study, a routine dental service was defined by claims for service codes 01 or 04 of the German uniform fee schedule for dental services; BEMA [42]. BEMA 01 is the pivotal code that describes a routine check-up investigation and can be reimbursed for each person once per semester (and dentist). BEMA 04 describes a periodontitis screening to check if treatment for periodontitis is indicated and can be reimbursed every two years. Thus, while mostly describing preventive check-ups, *routine dental services* also encompass periodontal exams. The recommendation of the National Association of Statutory Health Insurance Dentists is at least one check-up per calendar-year [31]. While at least one routine dental service during the individual two-year baseline constitutes a liberal criterion for a regular dental service utilization, adherence to the recommendation of one check-up per calendar year is possible with only one routine dental service in an individual two-year period. We addressed this by applying a stricter criterion in a sensitivity analysis. For the control group, we used a substantially larger random sample selected according to the same criteria as the study group but without the onset of home care need in 2017 (see supplementary material). We drew index dates in the control group randomly with replacements from the index date distribution of the study group. Thus, each person was observed for a two-year baseline (starting in 2015) ending at their respective index date (in 2017) and a three-year follow-up (ending in 2020) that started at the index date.

**Fig. 1:**
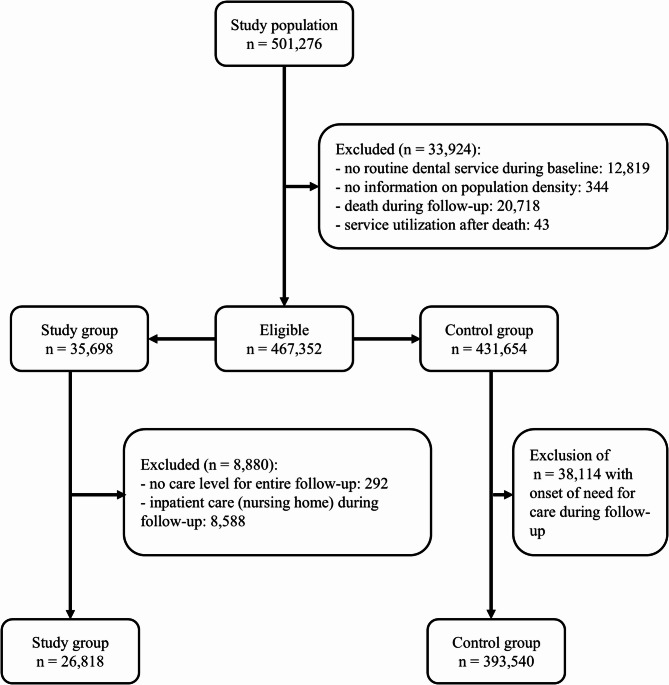
Flow-chart of the study population**.** The baseline and follow-up periods comprised individual time frames two years before and three years after the index date. The index date describes a date in 2017

We excluded all persons without population density information. Moreover, we excluded all persons who died during follow-up or for whom a health service (or new care level) was recorded more than one quarter after the recorded quarter of death (see supplementary material). We excluded persons in the study group whose care level was not recorded for the entire follow-up. Furthermore, we excluded persons who switched to inpatient care during follow-up. This included persons with more than 56 days in short-term nursing home care within a year because short-term care is only reimbursed for a maximum of 56 days per year [43]. Lastly, we excluded all persons in the control group with an onset of long-term care need during follow-up. Figure 1 displays the selection process and all selection criteria.

### Variables

We investigated the utilization of all dental services (irrespective of the recorded BEMA code) with the outcomes: any dental service use (binary) and the number of dental service cases during follow-up. A case defines a dentist visit and the respective treatment. If a treatment requires multiple visits, this is billed with the same case. The first visit within a calendar-quarter always defines a new case. As we only selected persons with utilization in the baseline, not using any dental service in the follow-up period might indicate a discontinuation of a presumably regular utilization. Because utilization has been shown to be increasingly symptom-driven for older persons [11] and persons with care dependency [44], we also investigated routine dental services. Thus, any routine dental service use (binary) and the number of routine dental services served as additional outcomes. As independent variable, we used the group assignment (study vs. control group). To test our secondary objective, we also used population density at the index date (low, medium, or dense) in an interaction analysis.

### Risk adjustment

Observational studies like this cannot utilize randomization and thus are at risk of imbalances in the covariate distributions between groups. If these covariates are also associated with the outcomes, they confound the association of group and outcome variables. Balancing relevant covariates like age [5, 6], gender [22], or prior utilization, addresses these differences. To account for baseline differences between groups, we applied entropy balancing [45] a reweighting algorithm that weights the individuals in the control group such that means, variance, and skewness of covariates closely match those in the study group. Entropy balancing can achieve more balanced covariate distributions than other common adjustment procedures [45, 46]. We used age, gender, number of dental (routine) services during baseline (proxies for prior utilization), days since the last dental (routine) service (proxies for prior utilization), and population density (proxy for regional variation [36]) at the index date. The estimated weights were used in all analyses if not stated otherwise. To test our secondary objective, we did not use population density as balancing variable, but applied entropy balancing within each stratum of population density to achieve balance between groups in each of them. These weights were only used in the interaction analysis.

In a sensitivity analysis, we additionally adjusted for medication-based comorbidities [47] present in ≥ 1% of both groups and healthcare costs in different health sectors during baseline (an indicator for healthcare utilization). As these variables might be related to functional status, we only used them in a sensitivity analysis investigating whether the onset of a home care need affects dental service utilization beyond overall healthcare utilization tendency and morbidity. A description of the risk adjustment variables can be found in the supplementary material.

### Statistical methods

To analyze the dental service utilization after the onset of home care, our main analysis was a comparison of study and control group using weighted logistic regressions for binary outcomes and weighted negative binomial regressions for count outcomes. To analyze the secondary objective of testing potential variations of the effects with regard to population density, we included population density and its interaction with the group variable into the respective models.

We conducted five sensitivity analyses to the main analysis (see supplementary material): First, we used a two-year follow-up to rule out any impact of the COVID-19 pandemic. Second, we selected all persons who survived for at least one year including all who died during the second or third follow-up year. Third, we used medication-based comorbidities and costs in all health sectors during baseline. Fourth, because recommendations to go to the dentist are based on calendar years [31, 32], not individual periods, we conducted an analysis based on calendar years using 2015–2016 as baseline and 2018–2020 as follow-up period. Here, we applied a stricter inclusion criterion selecting all persons with at least one routine service per baseline year. Fifth, we conducted an unweighted analysis to describe the impact of entropy balancing.

The main analysis and its sensitivity analyses are reported as risk ratios (logistic regressions) or rate ratios (negative binomial regressions). For the main analysis, we also report predictions in the respective group (probabilities or counts) and their differences. For the models for the secondary objective, those including interaction terms, we visualized the predictions for control and study group in the respective levels of population density to facilitate interpretation [48]. To access potential variations of the group differences with regard to population density, we calculated marginal effects between study and control group within each level of population density as well as differences in these differences.

Lastly, we conducted explorative analyses within the study group (without using entropy balancing weights), to investigate which factors were associated with the utilization of dental services among persons in need for home care. We used the variables: Age (in 5-year categories), gender, care level, use of professional home care, indications of wheelchair/scooter or walking aid use, and diagnosis of dementia during follow-up (see supplementary material). Entropy Balancing was conducted with Stata 16 (StataCorp, College Station, TX) and analyses with R (version 4.40). Because we tested the same hypothesis with four outcomes, we used α = 0.0125.

## Results

We included 26,818 persons in the study group and 393,540 persons in the control group. Characteristics of both groups are shown in Table 1 (supplementary Table 1 displays the stratified weighting for the interaction analysis). Before applying entropy balancing, the study group was older and utilized fewer dental services in the baseline period. Approximately 79% of the study group had care level 1–2 at the beginning of the follow-up.

**Table 1:** Descriptive statistics before and after entropy balancing

	Study group (*N* = 26,818)	Control group (*N* = 393,540)	
		Before EB	After EB
**Characteristics during baseline (2 years)**			
Female [%]	71.9	66.7	71.9
Age [mean, (SD)]	79.7 (7.5)	71 (7.2)	79.7 (7.5)
Days since last dental service [mean, (SD)]	191.2 (159.5)	152.6 (138.3)	191.2 (159.5)
Days since last routine dental service [mean, (SD)]	230.9 (170.3)	187.3 (151.1)	230.8 (170.3)
Dental services [mean, (SD)]	4.4 (2.8)	4.6 (2.7)	4.4 (2.8)
Routine dental services [mean, (SD)]	2.3 (1.1)	2.5 (1.1)	2.3 (1.1)
Medium population density [%]	44.7	45.6	44.7
Dense population density [%]	38.4	34.7	38.4
**Care level at the beginning of follow-up**			
Level 1 [n, (%)]	6,602 (24.6)		
Level 2 [n, (%)]	14,473 (54)		
Level 3 [n, (%)]	4,842 (18.1)		
Level 4 [n, (%)]	777 (2.9)		
Level 5 [n, (%)]	124 (0.5)		

*EB* Entropy Balancing; *SD* Standard deviation; all values were rounded to the first digit

**Fig. 2:**
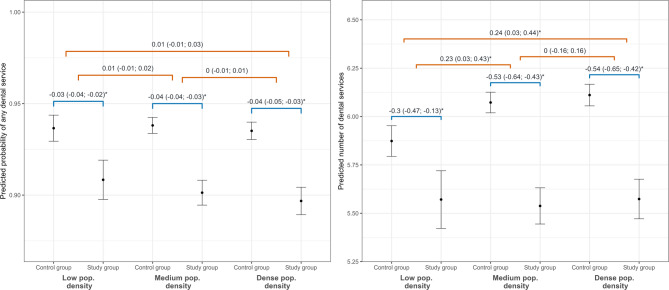
Results from generalized linear regressions with interaction with population density (secondary objective). Both figures depict predictions from the respective models (logistic regression left; negative binomial regression right). Horizontal lines in blue color (bottom) indicate differences within each level of population density while horizontal lines in red orange color (top) indicate differences in these differences. 98.75% confidence intervals built with robust standard errors are depicted in parentheses. This figure describes the outcomes any dental service use and number of dental services uses. See supplementary Fig. 1 for a similar figure for routine dental services. * Confidence interval does not include zero

The results of the interaction analysis of need for home care and population density to address our secondary objective can be found in Fig. 2 and supplementary Fig. 1. The probability for using any dental service and the number of utilizations were lower in the study than the control group in each level of population density, although the difference between groups in the number of services was smaller among persons living in areas with low population density. The analysis of routine dental services showed similar results (supplementary Fig. 1 ). As our primary focus was the onset of need for home care and both groups differed across all levels of population density in all outcomes, we conducted all further analyses without the interaction with population density.

**Fig. 3:**
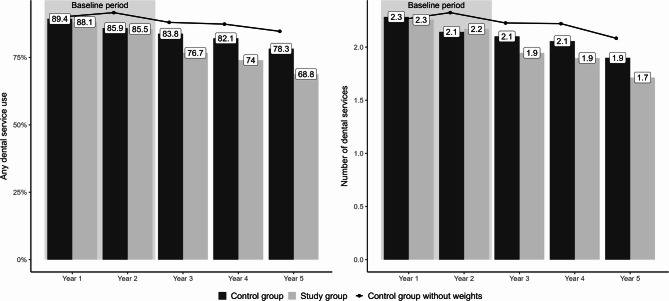
Utilization of dental services per year (all dental services)

Figure 3 shows the utilization per study year considering all dental services. Descriptively, the differences between both groups slightly increased over the follow-up.

Table 2 shows the results of the main analysis. Overall, 6.3% in the control and 9.9% in the study group did not utilize any dental service. No routine dental service was utilized by 7.7% in the control and 12.8% in the study group.

**Table 2:** Results of weighted generalized linear regressions (main analysis)

Outcome	Prediction study group	Prediction control group	Difference	Ratio
**All dental services**				
Any dental service^a^	0.9 (0.9; 0.91)	0.94 (0.93; 0.94)	−0.04 (−0.04; −0.03)	0.96 (0.96; 0.97)
Number of dental services^b^	5.56 (5.49; 5.62)	6.06 (6.02; 6.09)	−0.5 (−0.57; −0.43)	0.92 (0.91; 0.93)
**Routine dental services**				
Any routine dental service^a^	0.87 (0.87; 0.88)	0.92 (0.92; 0.93)	−0.05 (−0.06; −0.05)	0.94 (0.94; 0.95)
Number of routine dental services^b^	2.79 (2.76; 2.82)	3.27 (3.25; 3.28)	−0.48 (−0.51; −0.44)	0.85 (0.85; 0.86)

^a^Values were calculated with logistic regression; ^b^Values were calculated with negative-binomial regression

Parentheses depict 98.75% confidence intervals built using robust standard errors. All values were rounded to the second digit

Analogous to the main results, the sensitivity analyses showed a lower dental service utilization in the study group across all analyses and outcomes (supplementary Table 2 and 3). While the risk ratios in the calendar-year-based analysis were the least pronounced across sensitivity analyses, there also was a higher overall probability for any dental or any routine service in both groups (any dental service: 93.1% in the control, 97.4% in the study group). Analyses without using entropy balancing weights produced larger differences than the main analysis. Lastly, the explorative analyses (supplementary Table 4) within the study group showed that being aged 90–94, a higher care level (≥ level 3), professional home care, records indicating a wheelchair/scooter, and dementia were consistently associated with a lower utilization of dental services across outcomes. A recorded walking aid use was associated with a higher utilization across outcomes.

## Discussion

In this observational study with statutory health insurance claims data from Germany, we investigated the utilization of dental services after the onset of need for home care in persons aged ≥ 60 years. We compared this study group with a control group without need for long-term care balanced with entropy balancing. Persons in both groups utilized at least one routine dental service in the two-year baseline. Across outcomes and levels of population density, we obtained a lower utilization of dental services in the study group. A higher proportion of persons with a need for home care than of persons in the control group did not use any dental service during the 3-year follow-up and thus discontinued a previously presumably regular dental service utilization.

In line with literature, persons in need of long-term home care were less likely to utilize dental services than those without the need for home care [22, 24]. This difference was slightly more pronounced for routine dental services, which might emphasize that utilization becomes symptom- rather than check-up-driven in older adults [11]. After the onset of a need for home care, persons discontinued to use dental services within 3 years more often than those who did not become care-dependent. This expands the results of Czwikla et al. [22] who compared older adults in the year before and after transitioning to home care. Notably, the differences between the utilization of dental services in home care versus no care dependency were more pronounced than in our analysis. However, our study group had a higher proportion of care levels 1–2 than the home care recipient group of Czwikla et al. [22]. Hence, our study group may had a higher functional status, which likely increased the probability to utilize dental services. Additionally, we only selected persons with prior utilization to investigate a potential discontinuation which might explain why we obtained a high yearly utilization of dental services in both groups (Fig. 3) compared to studies from Germany [22, 25, 49], Europe [50], Australia [51], or the USA [52]. In line, the proportion of persons with dental service use was even higher when we applied a stricter criterion for a regular prior utilization in the calendar-year-based sensitivity analysis. Overall, our sample likely reflected persons for whom regular dentist visits were rather important and the study group had a relatively high functional status. However, even in this sample the onset of need for home care affected dental service utilization.

Multiple reasons might explain why the onset of a home care need was associated with a lower utilization of dental services. Besides factors related to the care need itself like frailty [53] and thus reduced functional ability, other factors might have affected the group differences. Fewer dental services might have been utilized in the study group because more persons in this group might have been edentulous. Henni et al. [30] argue that despite a reduction of edentulousness in the general population, it is still high for persons receiving home health care services. Although regular dentist visits are also recommended for persons with complete dentures [54, 55], the perceived treatment need might be lower for edentulous persons [23]. Accordingly, several studies have shown that being edentulous decreases the utilization of dental services [9, 56]. Thus, differences between study and control group might at least partly be explained by differences in edentulousness and perceived treatment need.

The interaction analyses with population density to address the secondary objective showed that the utilization of dental services was lower in the study than the control group in all population density levels. We obtained slightly smaller differences for persons living in an area with a low population density regarding the number of dental services and routine services. To a substantial part these originated from a lower utilization in the control group in low population density rather than differences across population density levels in the study group. Overall, the effect of population density was rather small. However, it might not be the only regional factor affecting utilization among those with need for home care. For example, studies showed differences in utilization between the German East (former German Democratic Republic) and West [22, 49] or potential associations of utilization with dentist density [5].

Factors associated with a decreased utilization in the explorative analyses are in line with existing evidence: Very high age [5, 22], higher care levels [22, 24], professional home care [24], or a dementia diagnosis [22, 57]. Indications for a wheelchair/scooter were associated with a decreased utilization, supporting the role of impaired mobility [58, 59]. Notably, a recordindicating a walking aid was associated with a higher utilization. Nevertheless, the explorative nature of these analyses demands further investigations.

### Strength & limitations

A strength of this study was the three-year follow-up. Thus, we could investigate whether the onset of home care affected dental service utilization beyond the following year in which dental services might not be a priority as care-dependent persons have to adjust to a new situation. Furthermore, investigating the number of utilized dental services and routine services provided a more nuanced picture than only investigating whether a dentist was visited or not. We only included persons with routine dental service use during the baseline. This implies more persons with need for home care might have *discontinued* to utilize dental services. While at least one recorded routine dental service in the baseline period does not necessarily imply a regular service utilization, applying a stricter definition (≥ 1 routine service per calendar-year) in a sensitivity analysis produced similar results. Another strength of this study was the large sample size, which allowed a precise estimation. However, in large samples even tiny differences can be distinguished and if the differences found here are of practical relevance remains to be evaluated.

A limitation was the operationalization of routine dental services. Recorded check-ups might have been provided when the reason for visiting the dentist was a symptom rather than a check-up. Moreover, a balanced sample could only be achieved by either disregarding health status and health service costs in the risk adjustment or by assigning high weights to a few individuals in the control group. While the results were robust across sensitivity analyses, we possibly did not access some aspects of morbidity such as the severity of the chronic conditions. Likewise, potentially relevant regional information like dentist density [5] or federal state [22, 49] were not accessible. Additionally, some of the variables used in the explorative analyses were only roughly approximated (e.g., w*heelchair/scooter* or *professional home care*).

Unfortunately, the health insurance claims data do not contain measurements like the DMF-T (decayed, missing, and filled teeth) or other indices that indicate the oral health status. However, oral health of persons with long-term care need has been shown to be worse than in the general population [25]. Thus, it is unlikely that a lower dental service utilization in the study group can be explained by a lower need for oral health care. 

Using data from a single health insurance limits the generalizability of our findings [60]. Despite a free choice of statutory health insurances in Germany, there are differences between health insurances populations especially with regard to socio-economic status [60]. However, how exactly these differences affect the generalizability of our results is unclear. Both groups had a constant care status (with need for home care and without need for long-term care) for the entire follow-up. This possibly led to a high overall utilization as those who are about to transition into long-term care or from home care to inpatient care were excluded but might have a particular low functional status and thus a particularly low dental service utilization. Likewise, excluding persons who died during the follow-up might have excluded those who were particularly prone for a low dental service utilization. Selecting these particular groups limits the generalizability but was necessary as the focus was on persons with a *lasting* need for home care. Lastly, excluding those without any dental service utilization prior to the onset of a home care need might have excluded those with the highest need for dental services.

## Conclusion

For older persons after the onset of a need for home care, the utilization of dental services was lower compared to persons without need for long-term care although both groups had a prior record of dental service use. This difference remained when further balancing for several chronic conditions and healthcare costs. In line with and expanding on previous literature, this study highlights the need to improve dental care to maintain a regular dental service utilization after the onset of home care.

## Supplementary Information

Supplementary Material 1.

## Data Availability

The datasets supporting the conclusions of this article are owned by the German statutory health insurance DAK-Gesundheit. In this case, anonymous data were used. For data availability, researchers must conclude a contract with the statutory health insurance. The licensee is permitted to use the data for the purpose of the research proposal within their company, exclusively. Licensees are not allowed to pass the data to a third party, or to create software or databases except for scientific publications.

## Abbreviations

BEMA: *Bewertungsmaßstab für zahnärztliche Leistungen* (Fee schedule for dental services)
InSEMaP: Interaction of Systemic Morbidity and Oral Health in Ambulatory Patients in Need of Home Care
DAK-G: Deutsche Angestellten Krankenkasse-Gesundheit

## References

[1] United Nations, Department of Economic and Social Affairs‚ Population Division . World Population Prospects 2024: Summary of Results (UN DESA/POP/2024/TR/NO. 9). 2024. New York: United Nations.

[2] Federal Statistical Office of Germany. 15. koordinierte Bevölkerungsvorausberechnung https://www.destatis.de/DE/Themen/Gesellschaft-Umwelt/Bevoelkerung/Bevoelkerungsvorausberechnung/begleitheft.html?nn=208696#ver%C3%A4nderung (2022). Accessed 20 December 2024.

[3] Jordan AR, Micheelis W. DMS V Core Results. In: Jordan AR, Micheelis W, editors. Fünfte Deutsche Mundgesundheitsstudie (DMS V). Cologne: Deutscher Ärzteverlag; 2016. p. 33–5.

[4] Gil-Montoya JA, Ferreira de Mello AL, Barrios R, Gonzalez-Moles MA, Bravo M. Oral health in the elderly patient and its impact on general well-being: a nonsystematic review. Clin Interv Aging. 2015;10:461–7. 10.2147/CIA.S54630. 10.2147/CIA.S54630 PMC4334280 25709420

[5] Schwendicke F, Krasowski A, Gomez Rossi J, Paris S, Kuhlmey A, Meyer-Lückel H, et al. Dental service utilization in the very old: an insurance database analysis from northeast Germany. Clin Oral Investig. 2021;25(5):2765–77. 10.1007/s00784-020-03591-z. 32995975 10.1007/s00784-020-03591-z PMC7524568

[6] McKenzie KW, Goodwin M, Pretty I. NHS dental service utilisation and social deprivation in older adults in North West England. Br Dent J. 2017;223(2):102–7. 10.1038/sj.bdj.2017.624. 28729568 10.1038/sj.bdj.2017.624

[7] Slack-Smith L, Hyndman J. The relationship between demographic and health-related factors on dental service attendance by older Australians. Br Dent J. 2004;197(4):193–9. 10.1038/sj.bdj.4811571. 15375412 10.1038/sj.bdj.4811571

[8] Grönbeck-Linden I, Hägglin C, Petersson A, Linander PO, Gahnberg L. Discontinued dental attendance among elderly people in Sweden. J Int Soc Prev Community Dent. 2016;6(3):224–9. 10.4103/2231-0762.183101. 27382538 10.4103/2231-0762.183101 PMC4916796

[9] Manski R, Moeller J, Chen H, Widström E, Listl S. Disparity in dental attendance among older adult populations: a comparative analysis across selected European countries and the USA. Int Dent J. 2016;66(1):36–48. 10.1111/idj.12190. 26465093 10.1111/idj.12190 PMC4728006

[10] Komulainen K, Ylöstalo P, Syrjälä A-M, Ruoppi P, Knuuttila M, Sulkava R, et al. Determinants for preventive oral health care need among community-dwelling older people: a population-based study. Spec Care Dentist. 2014;34(1):19–26. 10.1111/scd.12021. 24382367 10.1111/scd.12021

[11] Nitschke I, Stillhart A, Kunze J. Utilization of dental services in old age. Swiss Dent J SSO. 2015;125(4):433–47. 10.61872/sdj-2015-04-03. 10.61872/sdj-2015-04-03 26169279

[12] Strömberg E, Hagman-Gustafsson ML, Holmén A, Wårdh I, Gabre P. Oral status, oral hygiene habits and caries risk factors in home-dwelling elderly dependent on moderate or substantial supportive care for daily living. Community Dent Oral Epidemiol. 2012;40(3):221–9. 10.1111/j.1600-0528.2011.00653.x. 22070521 10.1111/j.1600-0528.2011.00653.x

[13] Aldossary A, Harrison VE, Bernabé E. Long-term patterns of dental attendance and caries experience among British adults: a retrospective analysis. Eur J Oral Sci. 2015;123(1):39–45. 10.1111/eos.12161. 25521216 10.1111/eos.12161

[14] Haag DG, Peres KG, Balasubramanian M, Brennan DS. Oral conditions and health-related quality of life: a systematic review. J Dent Res. 2017;96(8):864–74. 10.1177/0022034517709737. 28581891 10.1177/0022034517709737

[15] Algra Y, Haverkort E, Kok W, Etten-Jamaludin Fv, Schoot Lv, Hollaar V, et al. The association between malnutrition and oral health in older people: a systematic review. Nutrients. 2021;13(10):3584. 10.3390/nu13103584. 34684584 10.3390/nu13103584 PMC8541038

[16] Badewy R, Singh H, Quiñonez C, Singhal S. Impact of poor oral health on community-dwelling seniors: a scoping review. Health Serv Insights. 2021;14:1–19. 10.1177/1178632921989734. 10.1177/1178632921989734 PMC7841244 33597810

[17] Federal Statistical Office of Germany. Mehr Pflegebedürftige https://www.destatis.de/DE/Themen/Querschnitt/Demografischer-Wandel/Hintergruende-Auswirkungen/demografie-pflege.html (2024). Accessed 17 February 2025.

[18] Heinen I, van den Bussche H, Koller D, Wiese B, Hansen H, Schäfer I, et al. Morbiditätsunterschiede bei Pflegebedürftigen in Abhängigkeit von Pflegesektor und Pflegestufe. Z Gerontol Geriatr. 2015;48(3):237–45. 10.1007/s00391-013-0556-y. 24509639 10.1007/s00391-013-0556-y

[19] Rothgang H. Sicherung und Koordination der (zahn) ärztlichen Versorgung bei Pflegebedürftigkeit. In: Jacobs K, Kuhlmey A, Greß S, Klauber J, Schwinger A, editors. Pflege-Report. Stuttgart: Schattauer Verlag; 2017. p. 95–106.

[20] Brandhorst A, Focke K, Kalwitzki T, Müller R, Schmelzer C, Rothgang H. Versorgungspotentiale in der Mundgesundheit bei Pflegebedürftigen erkennen und nutzen. Gesundheits-und Sozialpolitik. 2016;70(3):53–8. 10.5771/1611-5821-2016-3-53.

[21] Göstemeyer G, Baker SR, Schwendicke F. Barriers and facilitators for provision of oral health care in dependent older people: a systematic review. Clin Oral Investig. 2019;23(3):979–93. 10.1007/s00784-019-02812-4. 30707299 10.1007/s00784-019-02812-4

[22] Czwikla J, Rothgang H, Schwendicke F, Hoffmann F. Dental care utilization among home care recipients, nursing home residents, and older adults not in need of long-term care: an observational study based on German insurance claims data. J Dent. 2023;136:104627. 10.1016/j.jdent.2023.104627. 37473830 10.1016/j.jdent.2023.104627

[23] Nitschke I, Majdani M, Sobotta BA, Reiber T, Hopfenmüller W. Dental care of frail older people and those caring for them. J Clin Nurs. 2010;19(13–14):1882–90. 10.1111/j.1365-2702.2009.02996.x. 20384671 10.1111/j.1365-2702.2009.02996.x

[24] Rothgang H, Müller R, Mundhenk R, Unger R. Zahnärztliche Versorgung Pflegebedürftiger. BARMER GEK Pflegereport 2014: Schwerpunkt: Zahnärztliche Versorgung Pflegebedürftiger. Siegburg: Asgard-Verlagsservice GmbH; 2014. p. 211–56.

[25] Nitschke I, Micheelis W. Krankheits-und Versorgungsprävalenzen bei Älteren Senioren mit Pflegebedarf. In: Jordan R, Micheelis W, editors. Fünfte Deutsche Mundgesundheitsstudie (DMS V). Cologne: Deutscher Zahnärzte Verlag DÄV; 2016. p. 557–78.

[26] Salmi R, Närhi T, Suominen A, Suominen AL, Lahti S. Perceived oral health and oral health behaviours among home-dwelling older people with and without domiciliary care. Gerodontology. 2022;39(2):121–30. 10.1111/ger.12542. 33565677 10.1111/ger.12542

[27] Schluter PJ, Askew DA, McKelvey VA, Jamieson HA, Lee M. Oral health among older adults with complex needs living in the community and in aged residential care facilities within New Zealand. J Am Med Dir Assoc. 2021;22(6):1177–83. e1. 10.1016/j.jamda.2020.06.041. 10.1016/j.jamda.2020.06.041 32736993

[28] Maille G, Bérengère S-S, Anne-Marie F, Ruquet M. Use of care and the oral health status of people aged 60 years and older in France: results from the National Health and Disability Survey. Clin Interv Aging. 2017;12:1159–66. 10.2147/CIA.S135542. 28814841 10.2147/CIA.S135542 PMC5546192

[29] Saunders R, Friedman B. Oral health conditions of community-dwelling cognitively intact elderly persons with disabilities. Gerodontology. 2007;24(2):67–76. 10.1111/j.1741-2358.2007.00160.x. 17518953 10.1111/j.1741-2358.2007.00160.x

[30] Henni SH, Skudutyte-Rysstad R, Ansteinsson V, Hellesø R, Hovden EAS. Oral health and oral health-related quality of life among older adults receiving home health care services: a scoping review. Gerodontology. 2023;40(2):161–71. 10.1111/ger.12649. 35943193 10.1111/ger.12649

[31] National Association of Statutory Health Insurance Dentists (KZBV). Das Bonusheft Spart bares Geld beim Zahnersatz https://www.kzbv.de/kzbv2022-pi-bonusheft-2.download.642b8262ebc2d1c002acb852a737f909.pdf (2024). Accessed 12 December 2024.

[32] Federal Ministry of Health. Zahnvorsorgeuntersuchungen https://www.bundesgesundheitsministerium.de/zahnvorsorgeuntersuchungen (2024). Accessed 20 December 2024.

[33] Czwikla J, Herzberg A, Kapp S, Kloep S, Schmidt A, Rothgang H, et al. Home care recipients have poorer oral health than nursing home residents: results from two German studies. J Dent. 2021;107:103607. 10.1016/j.jdent.2021.103607. 33607197 10.1016/j.jdent.2021.103607

[34] Friedrich A-C, Czwikla J, Schulz M, Wolf-Ostermann K, Rothgang H. Ärztliche Versorgung mit oder ohne Kooperationsvertrag? Eine Querschnittsuntersuchung in stationären Pflegeeinrichtungen in Bremen und Niedersachsen. Z Evid Fortbild Qual Gesundhwes. 2023;177:57–64. 10.1016/j.zefq.2022.11.011. 36964119 10.1016/j.zefq.2022.11.011

[35] Janssens L, Petrauskiene E, Tsakos G, Janssens B. Clinical and Subjective Oral Health Status of Care Home Residents in Europe: A Systematic Review. J Am Med Dir Assoc. 2023;24(7):1013-9.e40. 10.1016/j.jamda.2023.03.021. 37105236 10.1016/j.jamda.2023.03.021

[36] Reda SF, Reda SM, Thomson WM, Schwendicke F. Inequality in utilization of dental services: a systematic review and meta-analysis. Am J Public Health. 2018;108(2):e1–7. 10.2105/ajph.2017.304180. 29267052 10.2105/AJPH.2017.304180 PMC5846590

[37] Zimmermann T, Koenig A, Porzelt S, Schmage P, Konnopka C, Schellhammer S, et al. Interaction of systemic morbidity and oral health in ambulatory patients in need of home care (InSEMaP): an observational study at the sector boundary between dental and general practice care in Germany. BMJ Open. 2023;13(3):e063685. 10.1136/bmjopen-2022-063685. 36914197 10.1136/bmjopen-2022-063685 PMC10016254

[38] Benchimol EI, Smeeth L, Guttmann A, Harron K, Moher D, Petersen I, et al. The reporting of studies conducted using observational routinely-collected health data (RECORD) statement. PLoS Med. 2015;12(10):e1001885. 10.1371/journal.pmed.1001885. 26440803 10.1371/journal.pmed.1001885 PMC4595218

[39] DAK-Gesundheit. Kerndaten: Steckbrief Ihrer DAK-Gesundheit https://www.dak.de/dak/unternehmen/ueber-uns_12010 (2025). Accessed 09 May 2025.

[40] Federal Statistical Office of Germany. Current population https://www.destatis.de/EN/Themes/Society-Environment/Population/Current-Population/Tables/census-sex-and-citizenship-2024-basis-2022.html (2025). Accessed 09 May 2025.

[41] Federal Ministry of Health. Zahnärztliche Behandlung https://www.bundesgesundheitsministerium.de/zahnaerztliche-behandlung.html (2025). Accessed 9 May 2025.

[42] National Association of Statutory Health Insurance Dentists (KZBV). Einheitlicher Bewertungsmaßstab für zahnärztliche Leistungen gemäß § 87 Abs. 2 und 2h SGB V (BEMA): Anlage A zum Bundesmantelvertrag – Zahnärzte (BMV-Z) https://www.kzbv.de/wp-content/uploads/KZBV_BEMA_2025-01-01-1.pdf (2023). Accessed 19 May 2025.

[43] Federal Ministry of Health. Kurzzeitpflege https://www.bundesgesundheitsministerium.de/kurzzeitpflege (2024). Accessed 20 December 2024.

[44] Nitschke I, Hahnel S. Zahnmedizinische versorgung älterer menschen: Chancen und herausforderungen. Bundesgesundheitsblatt-Gesundheitsforschung-Gesundheitsschutz. 2021;64(7):802–11. 10.1007/s00103-021-03358-1. 34156484 10.1007/s00103-021-03358-1 PMC8241673

[45] Hainmueller J. Entropy balancing for causal effects: a multivariate reweighting method to produce balanced samples in observational studies. Political Anal. 2012;20(1):25–46. 10.1093/pan/mpr025.

[46] Matschinger H, Heider D, König H-H. A comparison of matching and weighting methods for causal inference based on routine health insurance data, or: what to do if an RCT is impossible. Gesundheitswesen. 2020;82(S 02):S139–50. 10.1055/a-1009-6634. 32066197 10.1055/a-1009-6634

[47] Huber CA, Szucs TD, Rapold R, Reich O. Identifying patients with chronic conditions using pharmacy data in Switzerland: an updated mapping approach to the classification of medications. BMC Public Health. 2013;13(1):1–10. 10.1186/1471-2458-13-1030. 24172142 10.1186/1471-2458-13-1030 PMC3840632

[48] Mize TD. Best practices for estimating, interpreting, and presenting nonlinear interaction effects. Sociol Sci. 2019;6:81–117. 10.15195/v6.a4.

[49] Rädel M, Bohm S, Priess H-W, Walter M. Zahnreport 2018: Schriftenreihe zur Gesundheitsanalyse. Siegburg: Müller Verlagsservice; 2018.

[50] Listl S, Moran V, Maurer J, Faggion CM Jr. Dental service utilization by Europeans aged 50 plus. Community Dent Oral Epidemiol. 2012;40(2):164–74. 10.1111/j.1600-0528.2011.00639.x. 21895735 10.1111/j.1600-0528.2011.00639.x

[51] Brennan DS, Luzzi L, Chrisopoulos S. Use of dental services among Australian adults in the National Study of Adult Oral Health (NSAOH) 2017–18. Aust Dent J. 2020;65(S1):S71-8. 10.1111/adj.12768. 32583584 10.1111/adj.12768

[52] Kramarow EA. Dental care among adults aged 65 and over, 2017. NCHS Data Brief. 2019;337:1–8. 31163014

[53] Janssens B, Tsakos G, De Visschere L, Verté D, De Witte N. Frailty as a determinant of dental attendance among community-dwelling older adults. Gerodontology. 2023;40(3):363–71. 10.1111/ger.12664. 36336964 10.1111/ger.12664

[54] Felton D, Cooper L, Duqum I, Minsley G, Guckes A, Haug S, et al. Evidence-based guidelines for the care and maintenance of complete dentures: a publication of the American College of Prosthodontists. J Prosthodont. 2011;20(s1):S1-12. 10.1111/j.1532-849X.2010.00683.x. 21324026 10.1111/j.1532-849X.2010.00683.x

[55] National Association of Statutory Health Insurance Dentists (KZBV). Zahnersatz – Therapien, Kosten und Beratung https://www.kzbv.de/kzbv2022-zahnersatz-de-web.media.38926fa765f81f5208fdb698bf4eb989.pdf (2022). Accessed 6 January 2025.

[56] Holm-Pedersen P, Vigild M, Nitschke I, Berkey DB. Dental care for aging populations in Denmark, Sweden, Norway, United Kingdom, and Germany. J Dent Educ. 2005;69(9):987–97. 10.1002/j.0022-0337.2005.69.9.tb03995.x. 16141084

[57] Lee KH, Wu B, Plassman BL. Dental care utilization among older adults with cognitive impairment in the USA. Geriatr Gerontol Int. 2015;15(3):255–60. 10.1111/ggi.12264. 24612371 10.1111/ggi.12264 PMC4145033

[58] Mariño R, Khan A, Tham R, Khew C-W, Stevenson C. Pattern and factors associated with utilization of dental services among older adults in rural Victoria. Aust Dent J. 2014;59(4):504–10. 10.1111/adj.12216. 25131698 10.1111/adj.12216

[59] Avlund K, Holm-Pedersen P, Schroll M. Functional ability and oral health among older people: a longitudinal study from age 75 to 80. J Am Geriatr Soc. 2001;49(7):954–62. 10.1046/j.1532-5415.2001.49187.x. 11527488 10.1046/j.1532-5415.2001.49187.x

[60] Hoffmann F, Koller D. Verschiedene regionen, verschiedene Versichertenpopulationen? Soziodemografische und gesundheitsbezogene Unterschiede zwischen Krankenkassen. Gesundheitswesen. 2017;79(01):e1–9. 10.1055/s-0035-1564074. 26492391 10.1055/s-0035-1564074

