# Supplementary material for: Utilization of dental services of older persons after onset of home care ­­– an observational study from the InSEMaP research project based on German insurance claims data
Source: BMC Geriatr. 2025 Oct 15;25:776. doi: 10.1186/s12877-025-06420-8 (PMC12523075; doi:10.1186/s12877-025-06420-8)
Supplement: Supplementary file 1 — Supplementary Material 1. [file 12877_2025_6420_MOESM1_ESM.pdf]

Supplementary material

Table of content

p. 3	**Deviations to the study protocol**
p. 3	**Approximating date of death**
p. 4	**Variables for risk adjustment**
p. 4-5	**Sensitivity analyses**
p. 5-7	**Additional variables used in explorative analyses**
p. 8	**Supplementary table 1**
p. 9	**Supplementary table 2**
p. 10	**Supplementary table 3**
p. 11	**Supplementary table 4**
p. 12	**Supplementary figure** 1
p. 13	**References**

Deviations to the study protocol

Notes on sample size

In contrast to the projections in the study protocol [1], in which we described that the size of the study group will be about n = 6000 and the control group will encompass at least five times as many persons, the realized samples were substantially larger. The reasons for this are twofold: First, the projections of the study group were based on a rather narrow definition of lasting need for home care. However, we decided that the DAK-G provides the data of all persons with an onset of a care need and we select those with a lasting need for home care according to the definition described in the methods section. Second, while validating the data delivery (i.e., before data preparation or analysis), we found substantial age differences between study and control group. To ensure a sufficient number of valid controls for applying the risk adjustment, we decided to select a control group about ten times the size of the study group.

Notes on selection criteria

In addition to the selection criteria implied by the inclusion criteria described in the study protocol ("no routine dental service during baseline", "death during follow-up", "no care-level for the entire follow-up", "transitioning to inpatient care", and "onset of need for care during follow-up [control group]"), we applied a few criteria to exclude implausible data. Consequently, we excluded all persons without information on the population density of their place of residency as well as all for whom health services were recorded after their death.

Approximating date of death

We only obtained information on the death date of the insured persons on a quarterly basis. Thus, to approximate a more precise death date, we used the last utilized health services within the quarter the death was recorded. Here, we used all health services sectors: inpatient or rehabilitation, outpatient, outpatient hospital, medication, medical devices, medical appliances, home healthcare services, dental services, or recorded care levels. For inpatient hospital stays, rehabilitation, and recorded care levels, we used the discharge or last recorded date as death date as we assumed that the death caused the inpatient or rehabilitation stay or the care level to end. For all other health services, we used the midpoint (i.e., central date) between the last utilized service and the end of the quarter in which a person died because we only knew that persons died at some point in between the last recorded health service and the end of the respective quarter. If no service was recorded within this quarter, we used the central date within that quarter as the death date.

Variables for risk adjustment

Population density of place of residency

The DAK-G provided information on whether the insured person lived in a region with low, medium, or dense population density based on information from the *Gemeindeverzeichnis* (list of municipalities) of the Federal Statistical Office [2]. The most recent version at the time of retrieval (May 9th, 2022) from the first quarter of 2022 was used. Using information from the *Deutsche Post,* a fine-grained list of zip codes was merged via the *Amtlicher Gemeindeschlüssel* (municipality key) to this information to later match these with the zip code in the DAK-G database. The linkage of this information to the claims data was performed by the DAK-G based on their regional information on the places of residency. For < 0.1% of all persons there was insufficient information to add the population density (n = 344 persons were excluded for this reason). We used the population density category recorded at the index date. We created two dummy variables with low population density as the reference category.

Medication-based comorbidities

We obtained 22 medication-based comorbidities using ATC-codes (Anatomical-Therapeutic-Chemical-codes [3]). Thus, a comorbidity was present if a drug with the corresponding ATC code was recorded during the baseline period.

Costs during baseline

We used the healthcare costs during baseline as an indicator for utilization of health services in one of our sensitivity analyses. To do this, we summed the costs during baseline in the following health sectors, respectively: outpatient, inpatient hospital, outpatient hospital, medication, medical appliances, medical devices, rehabilitation, home healthcare services, and dental services. Rare cases of negative values (due to administrative reasons) were set to zero. All costs were used at the date of the start of the treatment, calculated in 2020 Euro (i.e., transformed to the level of the Euro in 2020), adjusted for inflation using the Gross Domestic Product price index [4], and winsorized at the 99th percentile to mitigate the impact of extreme outliers.

Sensitivity analyses

The sensitivity analyses differed from the main analysis regarding the investigated time periods, the selection criteria, or variables used for risk adjustment. Here, we list all deviations to the main analysis of each sensitivity analysis.

Sensitivity analysis 1: Two-year follow-up

Due to the two-year follow-up we did not exclude all persons who transitioned to inpatient long-term care or died during the third follow-up year. Additionally, a continuous care level was an inclusion

criterion only for two years in the need for home care group. Persons in the control group with an onset of a care need also were only excluded if that happened within the first two years of follow-up.

Sensitivity analysis 2: Including patients who died during follow-up

The purpose of this sensitivity analysis was to evaluate the impact of only including persons who survived the entire follow-up in the main analysis. Thus, we relaxed the selection criteria and included all persons who deceased within year two or three of the follow-up period. However, as persons who died very shortly after becoming care dependent might have no prospect of visiting a dentist, we still excluded all persons who died in the first year of the follow-up. Applying these selection criteria led to a weighted mean follow-up time of 1057 days in the study and 1087 days in the control group. We calculated the proportion of the follow-up each person was observed and used it as offset variable in the negative binomial regressions to account for differences in observational length.

Sensitivity analysis 3: Medication-based comorbidities and baseline healthcare costs used in entropy balancing

For this sensitivity analysis, we applied the same selection criteria as in the main analysis. However, we adjusted for medication-based comorbidities and healthcare costs during the baseline in addition to the variables used in the main analysis.

Sensitivity analysis 4: Analysis based on calendar years

In this sensitivity analysis, we applied the criteria from the main analysis to a baseline from January 1, 2015 to December 31, 2016 and a follow-up from January 1, 2018 to December 31, 2020. Moreover, we applied a stricter selection criterion, only including persons with at least one recorded routine dental service in both baseline years. Lastly, we adjusted the variables used in the entropy balancing to the new baseline. Thus, we used the number of utilized (routine) dental services from 2015 to 2016. For defining the population density and the number of days since the last (routine) dental service, we used January, 1, 2017.

Sensitivity analysis 5: Without using entropy balancing weights

For this sensitivity analysis, we applied the same selection criteria and time periods as in the main analysis but did not use the entropy balancing weights in any analysis.

Additional variables used in explorative analyses

Age (categorized)

We used the age on December 31, 2017 and created 5-year categories. This led to the following categories: 60-64 years, 65-69 years, 70-74 years, 75-79 years, 80-84 years, 85-89 years, 90-94 years, ≥ 95 years.

Care level

We used a dummy-coded variable that indicates each person's care level during the follow-up. For this, we counted the days per each care level during follow-up. The variable referred to the care level with the highest number of days.

Professional home care

This variable describes whether a "*Pflegesachleistung"* (care benefits-in-kind) was recorded at least once during follow-up. These benefits-in-kind are used to compensate home care provided by professional care service providers. The variable disregards the amount of benefits-in-kind, whether any benefits in cash (*"Pflegegeld"*; to compensate care from informal caregivers) were claimed, as well as the time point during the follow-up of the first record of benefits-in-kind. Thus, it can only be seen as a rough approximation.

Wheelchair/Scooter

This variable describes whether a medical device with any "*Abrechungspositionsnummer*" (APN; Billing item number) that indicates the usage of a wheelchair or a scooter was recorded during follow-up. This included accessories to these devices. We used the following APN-codes ("x" represents any digit): 18 46 xx xxxx (indoor), 18 50 xx xxxx (indoor and outdoor/road traffic), 18 51 xx xxxx (road traffic), 18 65 xx xxxx (stairs), 18 99 xx xxxx (without special application location; additives). However, we excluded the following APN-codes as these relate to devices to be used by children: 18 46 01 5xxx, 18 46 01 6xxx, 18 46 07 xxxx, 18 50 03 1xxx, 18 50 03 2xxx, 18 50 03 6xxx, 18 50 05 xxxx, 18 51 04 xxxx. This selection was retrieved by manually searching a database of APN-codes, curated by the *GKV Spitzenverband* (The National Association of Statutory Health Insurance Funds [5]). It should be regarded as a rough proxy.

Walking aid

This variable indicates whether a medical device with an APN-code that indicates the usage of a walking aid was recorded during follow-up. This included accessories to these devices. We used the following APN-codes: 10 46 xx xxxx (indoor), 10 50 xx xxxx (indoor and outdoor/road traffic), and 10 99 xx xxxx (without special application location; additives). However, we excluded the following APN-codes as these relate to devices to be used by children: 10 46 02 3xxx, 10 46 03 xxxx. This selection was retrieved by manually searching a database of APN-codes, curated by the *GKV Spitzenverband* (The National Association of Statutory Health Insurance Funds [5]) for relevant codes. It should be regarded as a rough proxy.

Dementia

We identified the presence of dementia during follow-up using the following procedure: We used data from outpatient (only secure ("*gesichert")* and condition after ("Z*ustand nach"))*, inpatient hospital, outpatient hospital (only "secure" and "condition after" diagnoses), and medication records. Following Bauer et al. [6], we used the ICD-10-GM codes F00.-, F01.-, F02.-, F03.-, G30.- and the ATC-codes N06DA and N06DX01. Dementia was present if one of these ICD-10-GM codes was recorded by a hospital within the follow-up or a relevant medication (ATC-code) or outpatient diagnosis was recorded in at least two quarters within a year. If dementia was recorded in at least one of the follow-up years, we coded dementia as present during follow-up.

Supplementary table 1 Descriptive statistics in two-year baseline before and after applying EB in each level of population density

Variable	Study group	Control group before EB	Control group after EB
**Population density: Low**	**n = 4,518**	**n = 77,258**	
Female [%]	68	63.7	68
Age [mean, (SD)]	78.9 (7.3)	69.8 (7)	78.9 (7.3)
Days since last dental service [mean, (SD)]	195.4 (160.1)	154.7 (139.1)	195.4 (160.1)
Days since last routine dental service [mean, (SD)]	233.2 (170.5)	188.2 (151.3)	233.2 (170.5)
Dental services [mean, (SD)]	4.3 (2.7)	4.5 (2.6)	4.3 (2.7)
Routine dental services [mean, (SD)]	2.3 (1)	2.5 (1.1)	2.3 (1)
**Population density: Medium**	**n = 11,999**	**n = 179,547**	
Female [%]	71.3	66.6	71.3
Age [mean, (SD)]	79.9 (7.4)	71 (7.2)	79.9 (7.4)
Days since last dental service [mean, (SD)]	189.4 (159.1)	151.6 (137.8)	189.4 (159.1)
Days since last routine dental service [mean, (SD)]	229.9 (170.2)	186.6 (151)	229.9 (170.2)
Dental services [mean, (SD)]	4.4 (2.8)	4.6 (2.6)	4.4 (2.8)
Routine dental services [mean, (SD)]	2.3 (1.1)	2.5 (1.1)	2.3 (1.1)
**Population density: Dense**	**n = 10,301**	**n = 136,735**	
Female [%]	74.4	68.7	74.4
Age [mean, (SD)]	80 (7.6)	71.7 (7.3)	79.9 (7.7)
Days since last dental service [mean, (SD)]	191.5 (159.7)	152.5 (138.5)	191.4 (159.7)
Days since last routine dental service [mean, (SD)]	231.1 (170.5)	187.8 (151.1)	231.1 (170.5)
Dental services [mean, (SD)]	4.5 (2.8)	4.7 (2.7)	4.5 (2.8)
Routine dental services [mean, (SD)]	2.3 (1.1)	2.5 (1.1)	2.3 (1.1)

EB: Entropy Balancing; SD: Standard deviation; all values were rounded to the first digit.

Supplementary Table 2 Results from all sensitivity analyses

Outcome	Sensitivity analysis 1	Sensitivity analysis 2	Sensitivity analysis 3	Sensitivity analysis 4	Sensitivity analysis 5
Any dental service[a]	0.94 (0.94; 0.95)	0.95 (0.95; 0.96)	0.95 (0.95; 0.96)	0.96 (0.95; 0.96)	0.94 (0.93; 0.94)
Number of dental services[b]	0.92 (0.9; 0.93)	0.91 (0.9; 0.92)	0.89 (0.87; 0.9)	0.9 (0.89; 0.92)	0.85 (0.84; 0.86)
Any routine dental service[a]	0.92 (0.92; 0.93)	0.93 (0.92; 0.94)	0.94 (0.93; 0.95)	0.94 (0.93; 0.95)	0.91 (0.91; 0.92)
Number of routine dental services[b]	0.86 (0.85; 0.86)	0.84 (0.84; 0.85)	0.85 (0.83; 0.86)	0.84 (0.83; 0.85)	0.77 (0.76; 0.78)
	n_c = 416,004	n_c = 398,258	n_c = 393,540	n_c = 288,356	n_c = 393,540
	n_s = 32,424	n_s = 30,742	n_s = 26,818	n_s = 16,834	n_s = 26,818

[a] Analyzed with logistic regression (values depict risk ratios); [b] Analyzed with negative-binomial regression (values depict rate ratios).

Estimates and confidence intervals (98.75%) built using robust standard errors of study group (need for home care) versus control group. n_c: Sample size control group; n_s: Sample size in study group.

Sensitivity analysis 1: Two-year follow-up; Sensitivity analysis 2: Including those who died during the second or third year; Sensitivity analysis 3: Medication-based comorbidities and baseline healthcare costs used in entropy balancing; Sensitivity analysis 4: Analysis based on calendar years; Sensitivity analysis 5: Without using entropy balancing weights.

Supplementary Table 3 Results from all sensitivity analyses (log scale)

Outcome	Sensitivity analysis 1	Sensitivity analysis 2	Sensitivity analysis 3	Sensitivity analysis 4	Sensitivity analysis 5
**Any dental service**[a]					
Intercept	2.3 (0.01)	2.63 (0.02)	2.81 (0.04)	3.54 (0.03)	3.25 (0.01)
Study group	-0.5 (0.02)	-0.56 (0.03)	-0.61 (0.05)	-0.94 (0.04)	-1.05 (0.02)
**Number of dental services**[b]					
Intercept	1.41 (0)	1.79 (0)	1.84 (0.01)	1.88 (0)	1.88 (0)
Study group	-0.09 (0.01)	-0.1 (0)	-0.12 (0.01)	-0.1 (0.01)	-0.16 (0)
**Any routine dental service**[a]					
Intercept	2.08 (0.01)	2.42 (0.02)	2.59 (0.04)	3.32 (0.03)	3.01 (0.01)
Study group	-0.57 (0.02)	-0.65 (0.02)	-0.67 (0.04)	-1.02 (0.04)	-1.09 (0.02)
**Number of routine dental services**[b]					
Intercept	0.79 (0)	1.18 (0)	1.19 (0.01)	1.28 (0)	1.29 (0)
Study group	-0.16 (0)	-0.17 (0)	-0.17 (0.01)	-0.17 (0)	-0.26 (0)

[a] Analyzed with logistic regression; [b] Analyzed with negative-binomial regression

Estimates and robust standard errors (in parentheses). All values were rounded to the second decimal place; Sensitivity analysis 1: Two-year follow-up; Sensitivity analysis 2: Including those who died during the second or third year; Sensitivity analysis 3: Medication-based comorbidities and baseline healthcare costs used in entropy balancing; Sensitivity analysis 4: Analysis based on calendar years; Sensitivity analysis 5: Without using entropy balancing weights.

Supplementary Table 4 Results of explorative analyses within study group

Variable	Any dental service use (odds ratio)	Number of dental services (rate ratio)	Any routine dental service use (odds ratio)	Number of routine dental services (rate ratio)
(Intercept*)	11.23 (8.56; 14.72)	6.29 (5.92; 6.69)	8.95 (7.01; 11.42)	3.24 (3.08; 3.4)
Age: 65-69	1.14 (0.83; 1.57)	1.01 (0.94; 1.08)	1.25 (0.94; 1.67)	1.03 (0.97; 1.09)
Age: 70-74	1.32 (0.98; 1.78)	1 (0.93; 1.06)	**1.32 (1.01; 1.72)**	1.02 (0.97; 1.08)
Age: 75-79	**1.42 (1.09; 1.86)**	1 (0.94; 1.06)	**1.44 (1.13; 1.83)**	1.02 (0.97; 1.07)
Age: 80-84	1.28 (0.98; 1.66)	0.97 (0.91; 1.03)	**1.28 (1.01; 1.62)**	0.98 (0.94; 1.03)
Age: 85-89	0.95 (0.73; 1.23)	**0.86 (0.81; 0.91)**	0.9 (0.71; 1.14)	**0.87 (0.83; 0.92)**
Age: 90-94	**0.67 (0.51; 0.9)**	**0.8 (0.74; 0.86)**	**0.67 (0.51; 0.86)**	**0.8 (0.75; 0.85)**
Age: ≥ 95	0.65 (0.4; 1.04)	**0.72 (0.63; 0.83)**	**0.56 (0.37; 0.86)**	**0.73 (0.64; 0.82)**
Female	0.89 (0.8; 1.01)	0.97 (0.95; 1)	**0.89 (0.8; 0.99)**	**0.97 (0.95; 0.99)**
Care level 2	0.93 (0.82; 1.07)	1 (0.97; 1.03)	0.91 (0.81; 1.03)	0.99 (0.97; 1.01)
Care level 3	**0.83 (0.7; 0.98)**	**0.96 (0.92; 1)**	**0.75 (0.65; 0.87)**	**0.92 (0.89; 0.95)**
Care level 4	**0.66 (0.49; 0.88)**	**0.84 (0.78; 0.91)**	**0.56 (0.43; 0.72)**	**0.81 (0.75; 0.87)**
Care level 5	**0.45 (0.25; 0.79)**	**0.59 (0.48; 0.71)**	**0.28 (0.17; 0.46)**	**0.49 (0.4; 0.61)**
Professional home care	**0.73 (0.65; 0.81)**	**0.93 (0.91; 0.96)**	**0.73 (0.66; 0.81)**	**0.91 (0.89; 0.93)**
Wheelchair/Scooter	**0.86 (0.76; 0.96)**	**0.94 (0.91; 0.97)**	**0.82 (0.74; 0.91)**	**0.92 (0.9; 0.94)**
Walking aid	**1.14 (1.03; 1.27)**	**1.04 (1.02; 1.07)**	**1.13 (1.02; 1.24)**	**1.03 (1.01; 1.05)**
Dementia diagnosed	**0.85 (0.75; 0.96)**	**0.94 (0.91; 0.96)**	**0.86 (0.77; 0.96)**	**0.94 (0.92; 0.96)**

Estimates and 98.75% confidence intervals (in parentheses) per outcome; bold face indicates that the confidence interval does not include 1; * Reference categories: Age: 60-64, male gender, care level 1, no benefits-in-kind, no wheelchair/scooter, no walking aid, no dementia diagnosed in follow-up. Intercept values reflect odds and rates, respectively.

Supplementary figure 1. Results from the models with interaction with population density (routine dental services)

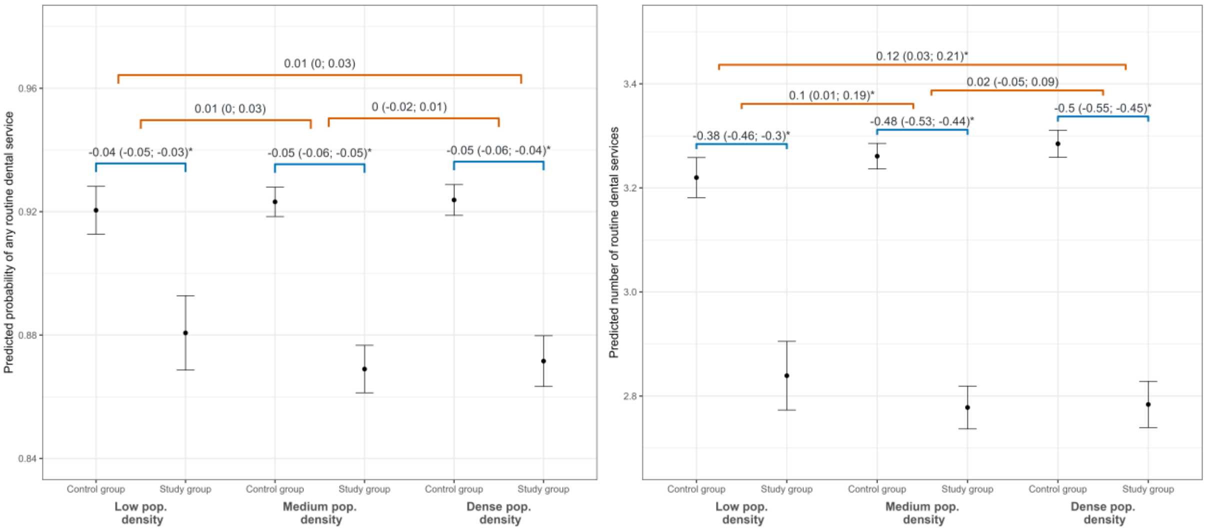

Notes. Both figures depict predictions from the respective models (logistic regression left; negative binomial regression right). Horizontal lines in blue color (bottom) indicate differences within each level of population density while horizontal lines in red orange color (top) indicate differences in these differences. 98.75% confidence intervals built with robust standard errors are depicted in parentheses. * Confidence interval does not include zero.

References

1. Zimmermann T, Koenig A, Porzelt S, Schmage P, Konnopka C, Schellhammer S, et al. Interaction of Systemic Morbidity and Oral Health in Ambulatory Patients in Need of Home Care (InSEMaP): an observational study at the sector boundary between dental and general practice care in Germany. BMJ open. 2023;13(3):e063685. https://doi.org/10.1136/bmjopen-2022-063685.
2. Federal Statistical Office of Germany. List of Municipalities Information System (GV-ISys) https://www.destatis.de/EN/Themes/Countries-Regions/Regional-Statistics/OnlineListMunicipalities/_inhalt.html#417212 (2022). Accessed 9 May 2022.
3. Huber CA, Szucs TD, Rapold R, Reich O. Identifying patients with chronic conditions using pharmacy data in Switzerland: an updated mapping approach to the classification of medications. BMC Public Health. 2013;13(1):1-10. https://doi.org/10.1186/1471-2458-13-1030.
4. Organisation for Economic Co-operation and Development. Economic References https://stats.oecd.org/Index.aspx?DataSetCode=HEALTH_ECOR (2024). Accessed 5 February 2024.
5. The National Association of Statutory Health Insurance Funds (GKV Spitzenverband). Hilfsmittel-verzeichnis https://hilfsmittel.gkv-spitzenverband.de/home (2024). Accessed 17 January 2024.
6. Bauer K, Schwarzkopf L, Graessel E, Holle R. A claims data-based comparison of comorbidity in individuals with and without dementia. BMC Geriatr. 2014;14:1-13. https://doi.org/10.1186/1471-2318-14-10.
